# Combined nutritional status and activities of daily living disability is associated with one-year mortality after hip fracture surgery for geriatric patients: a retrospective cohort study

**DOI:** 10.1007/s40520-024-02786-8

**Published:** 2024-06-08

**Authors:** Ying Chen, Ying Guo, Gang Tong, Yu He, Ruihua Zhang, Qi Liu

**Affiliations:** 1 Department of Geriatrics, Beijing Tongren Hospital, Capital Medical University, Beijing, 100730 China; 2 Department of Orthopedics, Beijing Tongren Hospital, Capital Medical University, Beijing, 100730 China

**Keywords:** Hip fracture, Prognosis, Nutritional status control, Activities of daily living, Older adults

## Abstract

**Objective:**

We aimed to explore the association combined nutritional status and activities of daily living disability with all-cause mortality of older adults with hip fracture in the first year after hospitalization.

**Methods:**

This is a single-center retrospective cohort study in older adults with hip fracture patients. Clinical data and laboratory results were collected from electronic medical record system of our hospital (2014–2021). The endpoint of this study was all-cause mortality in the first year after hospitalization.

**Results:**

A total of 303 older adults were enrolled and all-cause mortality was 21.8%. The study population was categorized by CONUT score. Patients in CONUT score 5–12 had a higher age, ASA status, CRP and creatinine level, more patients with history of fracture, pneumonia and delirium, meanwhile, lower BMI and ADL score, lower hemoglobin, lymphocyte, total protein, albumin, triglyceride, total cholesterol and one year survival than those in CONUT score 0–4 (all *P* < 0.05). Multivariable Cox analysis showed that BMI, ADL score and CONUT score were independent risk factors for all-cause mortality of hip fracture in older adults (HR (95% CI):2.808(1.638, 4.814), *P* < 0.001; 2.862(1.637, 5.003), *P* < 0.001; 2.322(1.236, 4.359), *P* = 0.009, respectively). More importantly, the combined index of CONUT and ADL score had the best predictive performance based on ROC curve (AUC 0.785, 95% CI: 0.734–0.830, *P* < 0.0001). Kaplan-Meier survival curves for all-cause mortality showed that patients with CONUT score increase and ADL score impairment had a higher mortality rate at 1 year compared to CONUT score decrease and ADL score well (Log Rank χ2 = 45.717, *P* < 0.0001).

**Conclusions:**

Combined CONUT and ADL score is associated with one-year mortality after hip fracture surgery for geriatric patients.

## Introduction

Due to population ageing, estimated hip fracture patients would grow exponentially worldwide, which is expected to be 2.6 million by 2025 and 6.3 million by 2050 [1, 2].

Hip fractures have a significant impact on the health of elderly people, and are known as the “last fracture in life” due to their high disability and mortality rates. About 35% of hip fracture survivors are unable to resume independent walking, and 25% of patients require long-term home care. The one-year mortality rate after fractures is as high as 20–30%, and medical expenses are expensive [3–5]. Surgery-related factors, including the delay of surgery, among others, and non-surgery-related factors, such as age, sex and BMI (body mass index), comorbidities, and special laboratory test results, were identified as risk factors [6]. Several predictive models were constructed based on these risk factors [7, 8]. However, the simplicity and practicality of the models for stratifying the mortality risk after hip fracture surgery was not satisfactory [9].

More and more evidence implicates that malnutrition, a crucial and modifiable risk factor, is associated with multiple unfavorable outcomes after hip fracture, such as increased risk of pneumonia, delirium, readmission, and mortality, etc. [10, 11]. In fact, nutritional intervention can help reduce complications after hip fracture surgery and improve daily living activities, which has been confirmed by randomized controlled trials or other high-level evidence [12, 13].

Meanwhile, the improvement of daily living activities is crucial for the outcomes of hip fractures, as individuals with decreased functional Status have an increased risk of disability and mortality [14–16].In addition, loss of mobility can lead to increased nursing costs and greater burden on the long-term care insurance system [17].

However, to our knowledge, existing research rarely has combined nutritional and functional status indicators as a composite parameter to explore and quantify the prognosis of patients with hip fractures.

This study aimed to explore whether combined nutritional status and activities of daily living disability is associated with one-year mortality after hip fracture surgery for geriatric patients.

## Materials and methods

### Study population

This was a single-center retrospective cohort study which was approved by the Clinical Research Ethics Committee of Beijing Tongren Hospital, Capital Medical University (TREC2023-KY026). A total of 303 older patients with a diagnosis of fragility (osteoporotic) hip fracture in our hospital from January 2014 to December 2021 were enrolled. Fragility hip fracture was defined as a hip fracture occurring after a minimal trauma, such as a fall from standing height or lower. Written informed consent was obtained from all patients. Clinical Trials.gov Identifier: NCT05814172.

The patients were divided into two groups according to Controlling nutritional status (CONUT) score: CONUT score 0–4 (*n* = 209) and CONUT score 5–12 (*n* = 94).

Inclusion criteria: (1) patients ≧ 65 years old; (2) patients diagnosed with hip fracture, including femoral neck fracture, femoral pertrochanteric fracture, and femoral subtrochanteric fracture; (3) patients whose injury was within 21 days of presentation; and (4)patients who had undergone surgery.

**Fig. 1:**
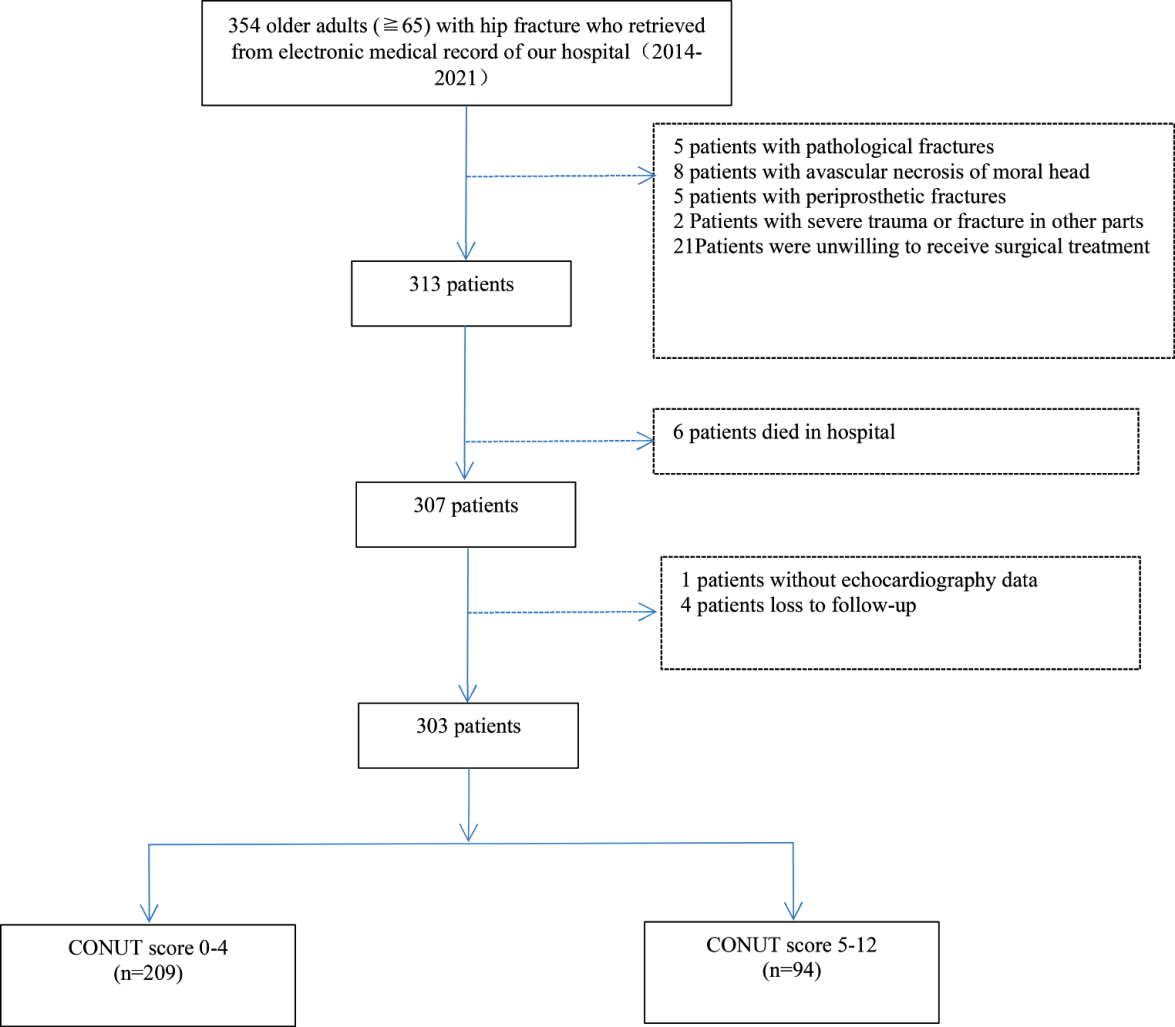
Flow of patients through the study

Exclusion criteria: (1) patients with pathological fractures due to tumor metastasis or infection or inherited bone disorder; (2) patients with avascular necrosis of femoral head; (3) patients with periprosthetic fractures; (4) patients with severe trauma or fracture in other parts; (5)patients died in hospital; (6) loss to follow-up. The flow of patients through the study was shown in Fig. 1.

### Variables and follow up

Variables extracted were divided in several groups, including demographic characteristics, comorbidities, complications, parameters related to surgery and laboratory parameters.

Demographic characteristics were age, gender and body mass index (BMI). Comorbidities included hypertension, coronary heart disease, diabetes, chronic kidney disease, cerebrovascular disease and previous fracture history. Postoperative complications included deep vein thrombosis (DVT), delirium and pneumonia which have a relatively high incidence rate. Functional independence status was evaluated by activities of daily living (ADL). Parameters related to surgery meaned American Society of Anesthesiologists (ASA) status. Laboratory parameters included hemoglobin (Hb), lymphocytes (Lyc), C-reactive protein (CRP), fasting blood glucose (Glu), serum creatinine (sCr), total protein (TP), albumin Alb), triglycerides (TG) and total cholesterol (TC). Those were collected from the electronic medical record system.

BMI was calculated as the weight in kilograms divided by the square of the height in metres, based on data from the medical records on admission. A BMI value of 18.5 kg/m^2^ was used as a cutoff for underweight, as suggested by the World Health Organization [18].

The ADL score assessment uses the Barthel Index, which is one of the most widely used tools assessing functional independence. The 10 performance items addressed by the Barthel index are presence or absence of fecal and urinary incontinence, and presence or absence of help needed with grooming, toilet use, feeding, transfers (i.e., from chair to bed), walking, dressing, climbing stairs, and bathing. The final score ranges from 0 (completely dependent patient) to 100 (totally independent patient) in 5-point intervals. The higher the score, the greater the activities of daily living. The ADL score assessment was performed for each patient before discharge by a trained nurse [19–22]. Severe functional impairment or dependency was defined as ADL score ≦ 50 based on previous literature.

ASA status graded physical status from 1 (normal) to 4 (severe systemic disease) [23].

In the present analysis, cut-off points of CRP, TP, TG were proposed by Youden’s index of ROC curve.

Anaemia is defined as a haemoglobin level two standard deviations below the normal for age and sex by the United Kingdom (UK) laboratory. This correlates as the following: Hb of men below 130 g/l, Hb of women below 120 g/l [24].

Our outcome was death from any cause within one year of surgery. After discharge, all enrolled patients were followed-up in an outpatients setting. Survival data were obtained via direct contact with patients or patients’ caregiver by their physicians at the hospital, or via telephone interview of their family by dedicated coordinators and investigators. The follow-up lasted for one year and the last follow-up was ended on December 31, 2022.

### Controlling nutritional status (CONUT) score

CONUT score was calculated from 3 variables: serum albumin concentration, total cholesterol concentration, and lymphocyte count. In this scoring system, point values are assigned to different ranges of the laboratory measures as follows: serum albumin score (1):≥35 g/L, 0 points; 34–30 g/L, 2 points; 29–25 g/L, 4 points; and < 25 g/L, 6 points; lymphocytes score (2):≥1.6*10^9^/L, 0 points; 1.2–1.59*10^9^/L, 1 point; 0.8–1.19*10^9^/L, 2 points; and < 0.8*10^9^/L, 3 points; and total cholesterol score (3): ≥4.65 mmol/L, 0 points; 3.62-4.64mmol/L, 1 point; 2.59-3.61mmol/L, 2 points; and < 2.59mmol/L, 3 points, CONUT score=(1)+(2)+(3). We defined normal and mild malnutrition as CONUT score 0–4 and moderate to severe malnutrition as CONUT score 5–12, as previously reported [25, 26].

### Statistical analysis

All continuous data were expressed in terms of the mean and the standard deviation of the mean or median and interquartile range (IQR) when not normally distributed. Normal distributional data were compared between two groups using independent-samples t test. The rank sum test was used to compare differences in nonnormally distributed data. Categorical data were expressed as frequencies and percentages and compared by the Chi-square. Cox proportional hazards regression models were used to identify patients at risk of all-cause mortality to calculate the multivariable-adjusted HRs and 95% CIs. Moreover, to check the predictive value of the model, a receiver operator characteristic (ROC) curve analysis was carried out. Kaplan-Meier survival curves and a log-rank test were used to compare mortality among risk-stratified groups. All analyses were performed using SPSS version 19.0 (SPSS, Chicago, IL, USA). A p value of < 0.05 was considered to be statistically significant.

## Results

### Baseline characteristics

As shown in Fig. 1, a total of 354 older patients with hip fracture were initially selected, and 51 were excluded according to the exclusion criteria. In the excluded patients, 21patients patients did not receive surgical treatment.

Finally, 303 eligible patients were included in this study, with a median age of 82 (78, 87) years, a predominance of female (69%), moderate to severe malnutrition rate of 31%. During one year follow up 66 patients (21.8%) died. The study population was categorized by CONUT score as follows: CONUT score 0–4, normal and mild malnutrition risk group (*n* = 209), and CONUT score 5–12, moderate to severe malnutrition risk group (*n* = 94). BMI could only be evaluated in 288 patients. Patients in CONUT score 5–12 had a higher age, ASA status, CRP and creatinine level, more patients with history of fracture, pneumonia and delirium, meanwhile, lower BMI and ADL score, lower hemoglobin, lymphocyte, total protein, albumin, triglyceride, total cholesterol and one year survival than those in CONUT score 0–4 (all *P* < 0.05), as shown in Table 1.

**Table 1:** Baseline characteristics of patients1

Characteristic	All patients	CONUTscore 0–4	CONUTscore 5–12	*P* value
	(*n* = 303)	(*n* = 209 )	(*n* = 94 )	
Demographics				
Age (y)	82(78–87)	81(75–86)	85(81–88)	< 0.001
Sex (male)	31(95)	32(66)	31(29)	0.899
Height (m)*	162(155–168)	160(155–168)	160(156–170)	0.981
Weight (kg)*	59(50–68)	60(50–70)	55(46–62)	0.012
BMI (kg/m^2^)*	22(19–24)	23(20–25)	21(18–23)	0.001
ADLscore	54(45–70)	60(48–75)	45(30–55)	< 0.001
ASAstatus 2/3/4% (n)	43(130)/50(152)/7(21)	47(99)/48(101)/4(9)	33(31)/54(51)/13(12)	0.006
Comorbidities % (n)				
HT	64.4(195)	66(138)	61(57)	0.365
CAD	35.0(106)	32(67)	42(39)	0.111
DM	30.4(92)	31(65)	29(27)	0.677
CKD	19.5(59)	17(36)	25(23)	0.141
CI	24.1(73)	25(53)	21(20)	0.442
History of fracture	28.4(86)	25(52)	36(34)	0.044
Complications % (n)				
pneumonia	10.6 (32)	7.7 (16)	17(16)	0.014
DVT	6.9 (21)	5.7 (12)	9.6 (9)	0.224
delirium	21.8(66)	18.2 (32)	29.8 (28)	0.024
Blood tests				
Hb, g/L	115(104–128)	122(110–130)	106(92–118)	< 0.001
Lyc, 10^9^/L	1.29(0.85–1.63)	1.37(0.97–1.76)	0.94(0.72–1.23)	< 0.001
CRP, mg/L	34.9(9.1–51.2)	20.3(4.75–39.9)	37.8(15.7–62.7)	< 0.001
Glu, mmol/L	7.3(5.7–7.9)	6.5(5.7–7.8)	6.4(5.7–8.1)	0.993
sCr, umol/L	79(57–88)	67(57–83)	76(58–93)	0.032
TP, g/L	64(58–69)	65(61–71)	58(55–62)	< 0.001
Alb, g/L	35 ± 4	37 ± 4	31 ± 3	< 0.001
TG, mmol/L	1.08(0.72–1.25)	1.02(0.81–1.34)	0.76(0.57–0.98)	< 0.001
TC, mmol/L	4.30 ± 0.98	4.6 ± 0.88	3.64 ± 0.87	< 0.001
1 year survival % (n)	78.2(237)	86.1(180)	60.6(57)	< 0.001

^1^ Continuous data are expressed as a median with IQR (25th to 75th centiles), and categorical data are expressed as % (n). Independent t tests and Mann-Whitney U tests were used to compare 2 continuous variables for normally and nonnormally distributed data. The χ2 test was used to compare proportions between groups.* with missing data. CONUT, controlling nutritional status; BMI, body mass index; ADL, daily of activity ability; ASA, American Association of Anesthesiologists; HT, hypertension; CAD, coronary artery disease; DM, diabetes mellitus; CKD, chronic kidney disease; CI, cerebral infarction; DVT, deep vein thrombosis; Hb, hemoglobin; Lyc, leukomonocyte; CRP, C-reactive protein; Glu, glucose; sCr, Serum creatinine; TP, total protein; Alb, albumin; TG, triglycerides; TC, total cholesterol

### Clinical outcomes and prognostic analysis

All patients were discharged and followed up for at least one year. All-cause 1-year-mortality was 21.8%. Univariable Cox proportional hazards analysis revealed that age (HR 2.876, *P* = 0.001), BMI (HR 3.194, *P* < 0.001), ADL score (HR 3.894, *P* < 0.001), with pneumonia (HR 2.099, *P* = 0.020), with delirium (HR 1.99, *P* = 0.008), Hb (HR 2.232, *P* = 0.001), CRP (HR 2.239, *P* = 0.001), TP (HR 3.432, *P* < 0.001), TG (HR 2.782, *P* < 0.001) and CONUT score (HR 4.361, *P* < 0.001) were significantly associated with all-cause mortality. Multivariable Cox proportional hazards analysis allowed us to identify a total of three independent predictive factors after adjusted for age, delirium, pneumonia, anemia, CRP, TP and TG. Being low BMI (HR 2.808, *P* < 0.001), having less ADL score (HR 2.862, *P* < 0.001) and higher CONUT score (HR 2.322, *P* < 0.001) were found to be statistically significant predictive factors of one year mortality after surgery (Table 2).

**Table 2:** Univariable and multivariable Cox analysis for the prediction of all-cause mortality1

Variables	univariate		multivariate	
	HR (95% CI)	*p* value	HR (95% CI)	*p* value
Age 65–80 year (referece)				
>80 year	2.876(1.568–5.277)	0.001		
BMI ≧ 18.5 kg/m2 (referece)				
<18.5 kg/m2	3.194(1.893–5.391)	< 0.001	2.808(1.638–4.814)	< 0.001
ADL score > 50 (referece)				
≦50	3.894(2.284–6.637)	< 0.001	2.862(1.637–5.003)	< 0.001
Pneumonia No (referece)				
Yes	2.099(1.123–3.924)	0.020		
Delirium No (referece)				
Yes	1.990(1.192–3.321)	0.008		
Hb non-anemia (referece)				
anemia	2.232(1.370–3.638)	0.001		
CRP ≦ 35 mg/L (referece)				
>35 mg/L	2.239(1.379–3.635)	0.001		
TP >64 g/L (referece)				
≦64 g/L	3.432(1.871–6.298)	< 0.001	2.002(0.999–4.015)	0.050
TG > 0.81mmol/L (referece)				
≦0.81	2.782(1.707–4.535)	< 0.001		
CONUT score 0–4 (referece)				
5–12	4.361 (2.482–7.661)	< 0.001	2.322(1.236–4.359)	0.009

^1^Univariate and multivariate analysis are performed using Cox’s regression model to identify meaningful prognostic factors. BMI, body mass index; ADL, daily of activity ability; Hb, hemoglobin; CRP, C-reactive protein; TP, total protein; TG, triglycerides; CONUT, controlling nutritional status

### ROC curve results

**Fig. 2:**
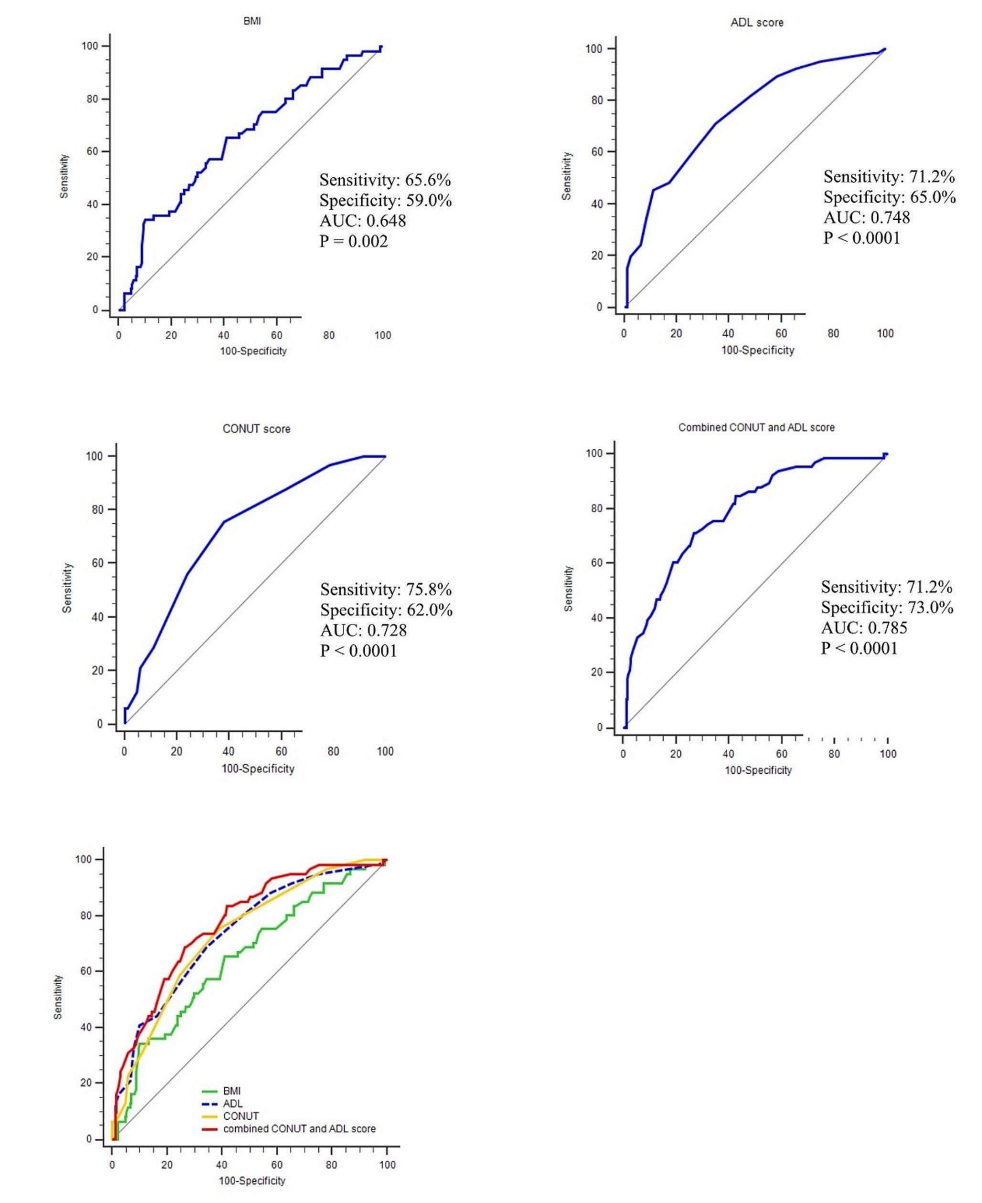
ROC Curve of the predictive model of all-cause mortality in patients with hip fractures. BMI, body mass index; ADL, daily of activity ability; CONUT, controlling nutritional status

The Fig. 2 showed that the the sensitivity, pecificity and AUC of ROC curve for BMI, ADL score and CONUT score predicting patients’ prognosis. When CONUT score was combined with ADL, the AUC was 0.785, with a sensitivity of 71.21% and a specificity of 73.00% (*P* < 0.0001), which was higher than for CONUT score, ADL score or BMI, a single characteristic (Fig. 2).

### Survival analysis results

**Fig. 3:**
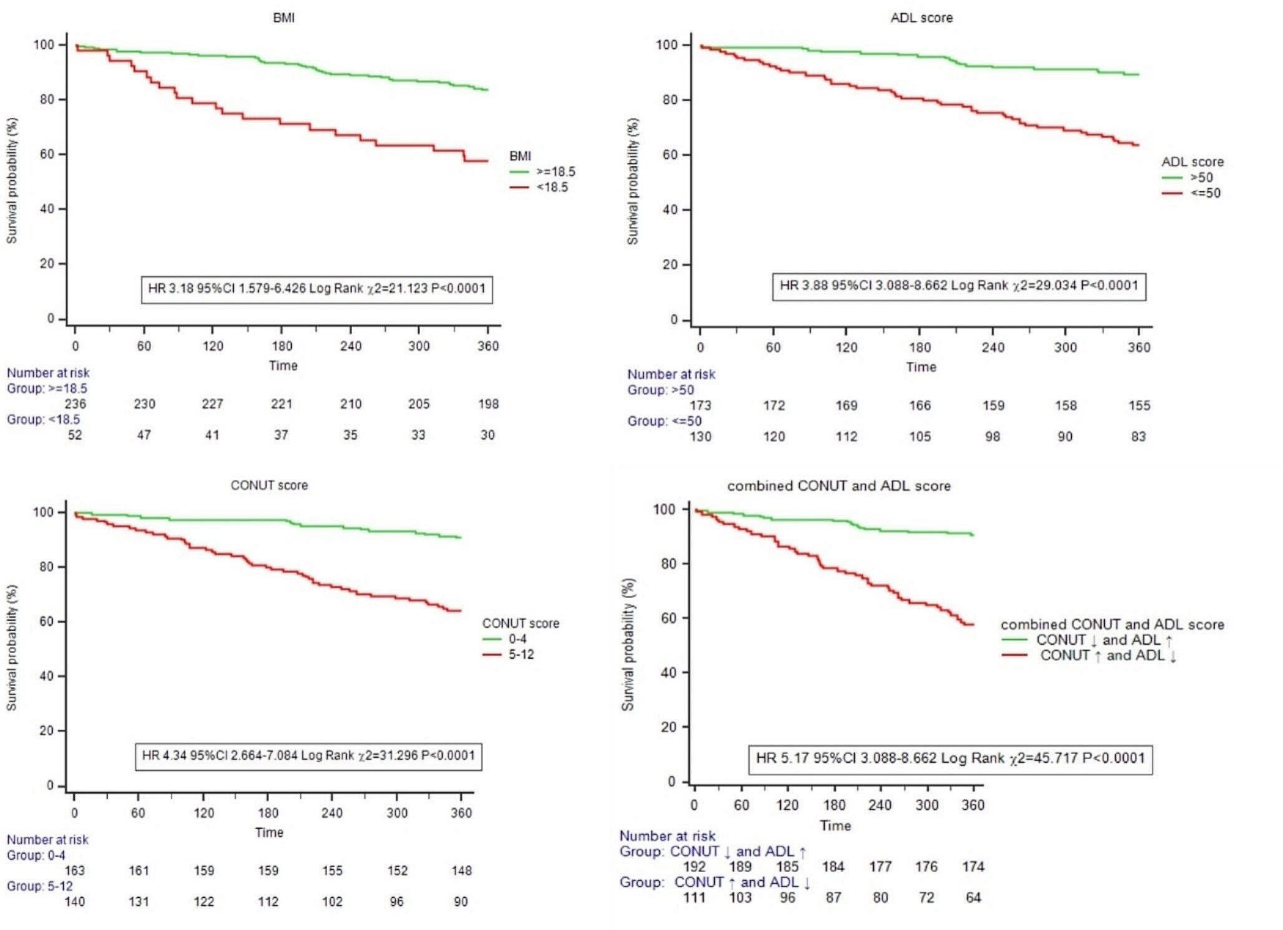
Kaplan–Meier estimates of all-cause mortality with patients stratified by different indicators Log-rank test was used to compare survival between groups. BMI, body mass index; ADL, daily of activity ability; CONUT, controlling nutritional status

Kaplan-Meier survival curves for all-cause mortality showed that patients with CONUT score increase and ADL score impairment had a higher mortality rate at one year compared to CONUT score decrease and ADL score well (HR 5.17, 95% CI 3.088–8.662, Log Rank χ2 = 45.717, *P* < 0.0001) (Fig. 3).

## Discussion

In this observational cohort study on the one-year mortality following hip fractures in older adults, we observed that combined index of CONUT score increase and ADL score impairment has good clinical value. To our knowledge, this study is the first exploring the relationship between a comprehensive index of nutritional status and autonomous mobility and mortality after hip fracture.

Firstly, high prevalence of malnutrition among older patients with hip fracture has been well documented, as well as its consequences on length of hospital stay, functional recovery, disability and in-hospital mortality [10, 27, 28]. This is because the nutritional status may further deteriorate after fracture surgery due to increased catabolism and reduced dietary intake. Even without the impact of surgery, malnutrition can lead to frailty and sarcopenia, which are common in elderly patients. Because muscles require sufficient protein and other nutrients to maintain their mass and function. Individuals with frailty may experience a decrease in muscle mass due to insufficient energy and nutrient intake, leading to the development of sarcopenia. Conversely, sarcopenia may also exacerbate frailty, as muscles are crucial tissues for maintaining daily activities and bodily functions. Frailty and sarcopenia affect the patient’s mobility and functional recovery. Malnutrition can also lead to a decline in the patient’s immune function, increase the risk of postoperative complications such as infection, resulting in prolonged hospital stays and increased mortality [29–31].

Considering the relevance of a correct identification of malnutrition in this population, the use of the most adequate screening tool should be warranted. In this study we used CONUT. Our study demonstrates the strong adverse impact of malnutrition on one year mortality in this population. The higher the CONUT score, the higher the risk of death within one year, consistent with previous reports [32, 33]. This information is important, because it allows clinicians to identify high risk patients, improve outcomes and healthcare efficiency and to target treatment to the most effective intervention.

More importantly, the combined index of ADL score and CONUT score had the best predictive performance. Due to the prolonged immobilisation required and to possible post-operative complications, hip fracture has a greater impact than other acute events on the functional decline of older patient. Therefore, on the contrary, the functional autonomy of older aduits reflects their postoperative prognosis well. In the study of 60,111 U.S. long-term nursing home residents, function declined substantially after fracture across all ADL score domains assessed, including transferring, mobility in bed, personal hygiene and toileting and increasing baseline ADL score dependence were all associated with decreased survival after hip fracture [34]. Ceolin’s study also suggested that functional loss in older adults hospitalised for proximal femur fractures was greatest in the first 6 months after discharge, and this increased the risk of death at 1 year [35]. However, malnutrition is related to losing walking independence (LWI) after hip fracture surgery. Cheng et al. evaluated the relationship between preoperative nutritional status assessed by CONUT score and postoperative 180 day walking independence in elderly Chinese patients with hip fractures. This study suggests that preoperative malnutrition is an important risk factor for postoperative loss of non independence in hip fractures, and nutritional screening at admission may have potential health benefits [36].

This study is the first exploring the relationship between a comprehensive index of nutritional status and autonomous mobility and mortality after hip fracture. A multidimensional assessment may be superior to considering a single aspect. CONUT, as an objective biomarker of nutritional status, although its detection method is simple, it reflects multiple physiological functions such as nutrition, inflammation, and immunity in the body, and complements the functional indicator ADL score to achieve complementary advantages. Our study clearly extends previous findings and reflects the role of multidisciplinary teams in postoperative rehabilitation of older fracture patients.

Indeed, there have been many developed nutrition screening tools, such as Mini Nutrition Assessment Short Form (MNA-SF), Nutrition Risk Screening 2002 (NRS 2002), and Subjective Global As assessment (SGA), which are often used in comprehensive assessment of older adults in recent years. While there are many screening tools available in the form of questionnaires, they heavily rely on subjective information provided by patients or their relatives. These tools can be influenced by subjective factors and compromised by cognitive impairments. Objective nutritional indicators are becoming a more and more frequently used tool in clinical work. Therefore, as a comprehensive nutritional scoring tool, CONUT score is an objective, easily obtainable and highly reproducible biomarker. Compared to other nutritional indicators, it may be more suitable for accurate nutritional assessment in fracture patients and provide stronger prognostic information for older adults fracture patients.

Secondly, except for CONUT and ADL score, this study also found that BMI was one of prognostic factors for all-cause mortality in elderly patients one year after surgery. BMI is a commonly available clinical parameter. Although pre-obesity and obesity as measured by BMI are associated with a higher risk of death in the general population, they are also associated with increased survival in various diseases, a phenomenon known as the ‘obesity paradox’, although its interpretation is a matter of debate. Some scholars believe that, the obesity paradox may be an artifact of selection bias for healthier patients in the preplanned surgical setting [37, 38]. However, in many studies analyzing survival in older adults, being overweight or obese has been found to be a beneficial factor. Theoretically, excess adipose tissue may provide for a metabolic reserve to be used in stress conditions, either chronic ones such as cancer or acute ones such as a fracture of a long bone and its surgical treatment. Overweight patients may thus be more tolerant of the internal and external stress that is coupled with traumatic injury and critical illness [39]. Consequently, a higher BMI could provide protection against acute muscle loss and, later on, frailty [40]. And underweight is the opposite, especially in old age, underweight status appears to be a stronger predictor for risk of death than is being overweight or obese [41, 42]. This study also supports that low BMI is an unfavorable factor for one-year overall mortality following hip surgery. However, in some older patients with spinal kyphosis or impaired cardiac and renal function, there may be difficulties in accurately measuring height and dry body weight, leading to potential deficiencies or errors in calculating BMI. This is a practical challenge.

This study has certain limitations. First, this was a single center, retrospective and observational study, which was subject to the clinical data of patients to a certain extent. The sample size of this study was limited, and the follow-up time was relatively short, which may cause a potential selection bias and center-specific to the research results. Secondly, weight refers to preoperative weight, and the acquisition of CONUT score is based on laboratory indicators at discharge. This study did not provide dietary information or changes in weight, CONUT score, and ADL score for each patient after discharge. Given the impact of hospital stay, postoperative ADL score and CONUT score may underestimate the patient’s condition. In order to clarify the answer to this question, further longitudinal research is needed.

## Conclusion

The combined index of CONUT score increase and ADL score impairment has good clinical value in assessing 1-year postoperative prognosis in older adults after hip fracture.

Going forward, intervention plans that use CONUT score as an objective detection indicator, ADL score as an outcome measure and BMI as a self-monitoring target may become practical standards for improving quality of life in geriatric patients with hip fractures after surgery.

## Data Availability

The data that support the fingdings of this study are available from the first author or cooresponding author upon reasonable request. This study has been registered on Clinical Trials.gov, and relevant data can be obtained from the website after the study is published.

## Declarations

### Research data (data sharing and collaboration)

There are no linked research data sets for this paper. The authors do not have permission.

to share data.
